# Perceived feasibility, acceptability and impact of the family involvement intervention for severe mental illness: a qualitative study in Masaka - Uganda

**DOI:** 10.1186/s13033-024-00634-w

**Published:** 2024-06-18

**Authors:** Andrew Kampikaho Turiho, Seggane Musisi, Racheal Alinaitwe, Elialilia S. Okello, Victoria Jane Bird, Stefan Priebe, Nelson Sewankambo

**Affiliations:** 1 Department of Psychiatry, Makerere University College of Health Sciences, Kampala, Uganda; 2 Mwanza Intervention Trials Unit, National Institute for Medical Research, Mwanza, Tanzania; 3 Unit for Social and Community Psychiatry, WHO Collaborating Centre for Mental Health Services Development, Queen Mary University of London, London, UK; 4 Department of Medicine, Makerere University College of Health Sciences, Kampala, Uganda

**Keywords:** Feasibility, Acceptability, Impact, Family involvement, Severe mental illness, Uganda

## Abstract

**Background:**

The burden of severe mental illness is high in low-resource settings like Uganda. But most affected people are not treated due to inadequacy of sectoral funding and trained mental health professionals. Medication has hitherto been the main method of treatment for severe mental illness worldwide. However, there is a growing realization that the use of community-based resource-oriented interventions like the family involvement are more effective and suitable for under-resourced settings. But there is a paucity of information about its applicability in Uganda.

**Methods:**

We based the intervention at the mental health unit of Masaka Regional Referral Hospital, involving 30 patients with SMI, 60 family members and friends, and 6 mental health clinicians. It was delivered through regular monthly meetings of 5 patients, 10 caretakers, and 2 clinicians each, for six months. A purposive sample of 15 patients, 15 caretakers, and 6 clinicians participated in this qualitative evaluation study after 6 months. Data was collected using in-depth interviews. Atlas.Ti (version 7.0.82) computer software was used in data analysis. Both priori and grounded codes were used to code data.

**Results:**

We evaluated perceived feasibility, acceptability and impact of the intervention in the Ugandan context. The findings were largely positive. Feasibility was mainly driven by: the training of group facilitators, field support and supervision, prior relationship between participants, and scheduling and timing of meetings. Acceptability was supported by: anticipation of knowledge about mental illness, process and content of meetings, safety of meeting environment, and choice of participants and venue. Impact was majorly in domains of: knowledge about mental illness, psychosocial aspects of mental illness, networking and bonding, and patients’ quality of life. The success of the intervention would further be enhanced by its decentralization and homogenized composition of groups.

**Conclusions:**

The intervention promises to spur improvement in the following main aspects of mental health services: accessibility since the meeting environment is more neutral and friendlier than the clinical setup; knowledge of mental illness; recognition of the important role of the family in management of mental illness; adoption of holistic approaches to mental illness; and quality of life of patients.

## Background

Severe mental illness (SMI) is long lasting. SMI is stigmatized; it severely compromises quality of life, family and other relationships, and educational and employment opportunities for the affected people, their families and communities [1, 2]. The prevalence of SMI in Uganda is estimated at 3% [3]. Despite this substantial burden, treatment rates for these disorders are low in low- to middle- income countries (LMICs) [4]. Up to 85% of people with SMI in LMICs receive no treatment compared to 35.5–50.5% in developed countries [3]. The main reasons for low access to treatment for people with SMI include a lack of trained mental health professions, and inadequate budget. According to the 2020 WHO country estimates [5], there are 2.57 mental health professionals per 100,000 population in Uganda compared to the global median of 13 per 100,000. Further, Uganda’s mental health sector receives 2.9% of the already meagre health sector budget [5]. This makes it difficult to adequately manage SMI or even provide the most basic mental health care to the majority of the population [6, 7]. This inadequacy results in late presentation of patients, long clinic queues, high clinician–patient ratios, long hospital stays, lost income and productivity, increased morbidity, and generally very poor prognosis [1, 8, 9].

There have been calls to implement low-cost, resource-oriented interventions in low-income countries to address the mental health treatment gap. Empirical evidence indicates that the modern comprehensive, community-based resource-oriented approaches such as the Family Psychosocial Involvement Intervention can be more effective compared to the psychiatric institution-based model of care and management of SMI [8, 10], and empowerment of individuals [11]. Such interventions have been shown to improve patient outcomes including: increasing medication adherence, preventing relapse and hospital re-admission, and shortening stays in hospital [12, 13]. However, to the best of our knowledge, no research has been conducted in Uganda to evaluate the family psychosocial involvement in the care and management of SMI.

The Family Psychosocial Involvement Intervention (FAPII) utilizes the family as an accessible resource that can be engaged through psycho-education, trialogue meetings, and family therapy to support patients with SMI [14]. Involving families in care appears essential to provide best possible support for patients and reduce stressful experiences of all family members, including the patient. As this is a low-cost intervention [15] that also uses existing family resources, it may be used to support effective community mental health care in Uganda and other similarly low-resourced settings. In this project, we aimed to refine family involvement intervention for the local context, and evaluate its perceived feasibility, acceptability, and impact amongst people with SMI, their caretakers and mental health clinicians in Uganda.

## Methods

### Study setting and design

Masaka Regional Referral Hospital is in Masaka City, located approximately 140 km, by road, south-west of Kampala. The mental health unit has 30 beds. Between 700 and 800 patients are seen at the facility each month and most of these are patients with SMI because those with mild mental illnesses usually seek alternative modes of treatment. The common SMIs seen at the facility include: Bipolar, Epilepsy (in the Ugandan system, most patients of Epilepsy are seen in psychiatric units), Depression, Schizophrenia, Alcohol and Substance Use Disorders, and HIV-related Psychosis. Data for this qualitative study was collected in 2020 as part of a larger mixed methods “open proof of concept study” aiming to test the feasibility, acceptability, and effectiveness of the FAPII in the Ugandan context. The main study, which is reported in a separate paper [16] (under review), compared patient and family caretaker outcomes at two sites – a control where patients received “Standard Care” and an intervention where patients in addition received the FAPII. The larger study involved a before and after intervention design whereby data was collected from both sites at baseline and at the end of the intervention. Data for this cross-sectional evaluation study, however, was only collected from the intervention site at the end of the six-month intervention. We used the Qualitative Impact Assessment Protocol (QUIP) that assesses the contribution of an intervention by relying on the perceptions of beneficiaries without statistical inference based on comparison to a control group [17].

### Inclusion and exclusion criteria

Patients and their family members or friends aged 18 to 65 years who did not intend to leave their areas of residence for the next six months were recruited from mental outpatient clinics at the Masaka hospital mental health unit. The inclusion criteria was broad and matched the majority of patients who are seen in community-based services. This would allow us to generalize the findings as well as compare to other studies which had been conducted globally assessing the effectiveness of the intervention. We included patients who; had a primary diagnosis of SMI (psychosis, bipolar disorder, psychotic/severe depression) assessed by the ICD-10, had received care/treatment for at least 6 months (duration of the intervention) at the facility, were able to provide informed consent (assessed by UBACC score of ≥ 14 after a maximum of 3 attempts), were able to communicate in Luganda (the language of intervention administration and main local dialect spoken in central Uganda) or English, and were able to identify their caregiver (family members or friends) that they would like to participate in the intervention with them, hereafter jointly referred to caretakers. Patients who had a primary diagnosis of substance-use disorder, organic psychosis and/or neurocognitive disorder were excluded because we wanted to focus on primary mental illness not mental illness secondary to other causes. We did not exclude people who had a secondary diagnosis of substance misuse, as long as it was comorbid with a primary eligible condition. Individuals with organic disorders and cognitive impairment were excluded as participants were required to be able to complete and understand a battery of assessment measures. Those who were in-patients at the time of recruitment were also excluded because they were considered too ill to participate. Moreover, since the intervention being tested was a community-based intervention, inpatients were excluded as they were not currently receiving community-based care, and would not be able to attend the community-based sessions. We included a caretaker living with the patient in one household and excluded that who did not have contact with the patient for the sake of continuous patient support and monitoring during the intervention. Mental health clinicians (psychiatric clinical officers, nurses, social workers, and occupational therapists) who were currently working at the outpatient clinic and had no plans to leave post within the next six months were included. We excluded clinicians who did not have clinical contact with the selected patients before and during the intervention.

### Intervention

FAPII meetings, also called psychosis seminars or trialogue meetings, bring together mental health professionals, patients with SMI, and their relatives in regular and open meetings to discuss and mutually learn about SMI. The meetings follow principles of ‘trialogue’ and emphasise the civil rights and strengths of both patients and their families. Evidence shows that the meetings can provide benefits for participants including opportunities to talk freely and to increase understanding of psychosis [18, 19]. In this study, the FAPII was implemented by clinicians, observed by the 2 research assistants (RAs), and supervised by senior researchers. FAPII was delivered as meetings organized based on the existing trialogue meeting guidelines which were adapted for purposes of the study. A pair of clinicians was responsible for 2 groups. To ensure consistency within meetings of each group, an attending clinician (the two, operating in turns) helped with group facilitation at each meeting. Groups assigned to a pair of clinicians met on different days to avoid clash of time tables for the clinicians. Each meeting was also observed by the 2 RAs. In all, the meetings involved 30 patients with SMI, 60 patients’ caretakers, and 6 mental health clinicians. These numbers were adopted from the main study where 30, the number of patients was determined based on recommendations regarding pilot and feasibility studies. Thirty (30) is seen as sufficient, based on central limit theorem [20], to be able to estimate variance and hence the parameters required for a full sample size calculation for a future trial. The numbers for caretakers and clinicians were similarly adopted; they were decided during the intervention development workshops with stakeholders and the criteria is described in the report, under review [16]. Participants in the FAPII were divided into 6 groups for purposes of the meetings. Each meeting, lasting for up to 2 hours with a break, brought together 5 patients, up to 10 caretakers (2 per patient), 2 clinicians, and 2 RAs (as observers) in regular monthly meetings for 6 months. The meetings were held at the local community center and were each chaired by an attending patient or caretaker, elected by the group members. The meetings provided a context for participants to learn through sharing experiences, mutual support and psychoeducation. Each meeting discussed pre-agreed, co-produced topics like causes of mental illness and how to improve livelihoods of families of persons with SMI.

### Procedures

Caseloads of the clinicians in the unit were screened by researchers with support from members of the clinical team, to identify potentially eligible patients. Eligible patients were given information about the study and invited to take part in the intervention by RAs. Patients who consented, were asked to identify up to two caretakers, whom they wished to attend the FAPII sessions with them. The identified caretakers were contacted by the research team using contact details provided by the patients. Interested caretakers (identified by RAs using phone calls) were given information about the intervention before meeting with the research team. The clinicians were approached and invited to take part in the intervention as facilitators of the meetings for which they were first trained by the researchers in a one-day workshop at the Masaka Hospital. Individuals met with a researcher to sign a consent form and complete a brief demographics form.

### Study population and sampling procedure

The population for the evaluation comprised of all the patients, their caretakers, and clinicians who were part of the family involvement group meetings. The qualitative sample size of 15 patients, 15 caretakers, and all the 6 clinicians was pragmatically considered to be enough for us to gain variance in experience. Based on their observations during the FAPII meetings, RAs purposively selected caregivers and patients they considered to have rich information needed to address the study objectives. To ensure maximum variation in the sample, deliberate effort was made to include both participants with positive and those with negative experiences of the intervention according to researchers’ observation. RAs further deliberately included a combination of participants who were always in attendance as well as those that missed some intervention meetings. Caretakers were also deliberately sampled to include different relationships with patient participants (e.g. parents, siblings, and friends), and those who took more and less of an active role in providing care for patient.

### Data collection methods and tools

Data was collected using in-depth interviews conducted by two RAs fluent in Luganda, were university graduates of psychology and social work, respectively, and were experienced in qualitative data collection. The topic guides for patients and care takers were translated into and administered in Luganda and the guide for clinicians remained in English. Interviews took place in a private room in the mental health unit that was designated for the research team during the research period. Topic guides were developed based on the study objectives, and the QUIP [17]. All the three categories of FAPII participants were asked about the following broad areas: how they found the family involvement groups (e.g. likes/dislikes, helpful/not helpful); if they thought that the family groups had had any impact on their (patients’ and caregivers’) lives and work (as a clinicians); factors that influenced participation in the meetings (e.g. reasons for attending/not attending); how they found the practical organization of the meetings; and how the FAPII meetings could be improved (e.g. best practice components). Clinicians, in addition, were asked about their experience with the group facilitation training, supervision, and the FAPII manual. Answers to the specific questions under each of these broad areas were analyzed for what they spoke to the study objectives. Each interview lasted for 40 to 60 min.

### Data management and analysis

Interviews were audio-recorded and then transcribed. Those in Luganda were translated into English. An external team with extensive experience in qualitative data management and proficiency in Luganda transcribed and translated the data under supervision of authors –TAK and AR. Data management and analysis were done by a team of three of the authors – TAK, AR, and OES as team leader. Analysis was done using Atlas.Ti (version 7.0.82). Using the framework method of data analysis [21], OES familiarized with the data in the transcripts, developed draft codes and discussed with the other authors before coding the data using a combination of both priori and grounded codes [22]. The codes were used to retrieve segments of the data and similar codes were grouped under themes. The identified themes and sub-themes were checked and refined. Memos describing the patterns and variations in the different segments of retrieved data were written. An inductive approach was used to provide new insights and richer understanding of the data without using preconceived categories. Verbatim quotations from the data were used to highlight key study findings.

## Results

Using data from a sample of 15 patients, 15 caretakers and 6 clinicians, we assessed the perceived feasibility, acceptability and impact of the intervention. These study objectives formed priori themes for data coding. We generated the results using the QUIP [17], previously described.

### Sociodemographic characteristics

Females were more represented both among patients (60%) and caretakers (60%). Patients’ age range was 25–64 years and the average age was 40.8. Caretakers’ age range was 21–67 years and the average age was 46.8. Most patients (53.3%) were single/separated. Caretakers were mainly single/separated (40%) and married (40%). The largest proportion of patients (40%) were unemployed/students and the largest proportion of caretakers (66.7%) were self-employed (refer to Table 1 below for more details).

**Table 1:** Distribution of participants by sociodemographic characteristics

SOCIODEMOGRAPHIC CHARACTERISTIC	PARTICIPANT CATEGORY	
	Patient (*N* = 15)	Caretakers (*N* = 15)
	n (%)	n (%)
**Sex**		
Male	6 (40)	6 (40)
Female	9 (60)	9 (60)
**Age (Years)**		
21–30	6 (40.0)	1 (6.7)
31–40	3 (20.0)	3 (20.0)
41–50	2 (13.3)	4 (26.7)
51–60	1 (6.7)	5 (33.3)
61–70	3 (20.0)	2 (13.3)
**Marital status**		
Single/ Separated	8 (53.3)	6 (40.0)
Married	6 (40.0)	6 (40.0)
Widowed	1 (6.7)	3 (20.0)
**Employment status**		
Salaried employment	3 (20.0)	0 (0.0)
Self employed	4 (26.7)	10 (66.7)
Unemployed/ student	6 (40.0)	1 (6.7)
Retired	1 (6.7)	1 (6.7)
Volunteer	1 (6.7)	2 (13.3

### Perceived feasibility of the family psychosocial involvement intervention (FAPII)

Data analysis yielded two sub-themes – perceived enablers for meeting facilitation, organization and participation; and perceived barriers for the meetings. The perceptions portrayed a feasible intervention since the perceived barriers were largely surmountable.

#### Perceived enablers for the meetings

The clinicians identified factors that made it easy for them to organize and facilitate the meetings. The timing of the meetings was considered convenient; the clinicians were allowed to exercise enough flexibility in facilitating the meetings and managing time. This enabled them to combine meeting facilitation with their regular clinical work. Clinicians also credited “adequate” facilitation of participants by the research team including the provision of transport refunds to the participants and availing refreshments during the meetings.

*The time frame was not all that bad and our leaders (chairs) were active. They would keep time and it was not all that very long, so it couldn’t affect our duties and on the financial part of it at least they were catering for us with drinks and bites so we were not stressed (during meetings). We got a transport refund so it made it comfortable for us ***(C)**.

Clinicians appreciated the group facilitation training they received prior to the intervention, which greatly enhanced their ability to successfully facilitate the meetings. They observed that the training was participatory and appropriate; and that trainers were competent, active and supportive throughout the intervention. Most clinicians, however, felt that the training duration of one day was too short.

*… Maybe we could have had the training for more than one day; because you know we are adults and sometimes when we sit for long we get tired and sometimes we are not able to grasp everything that is being taught ***(C)**.

The clinicians further rated highly the support and supervision received from the research team. They noted that the supervising team was very active; even when they were not available in person they usually followed up by phone and provided the necessary guidance. Clinicians also appreciated the supervision style; the supervisors provided ‘*just enough, non- stressing*’ supervision whereby they did not overwhelm clinicians with frequent and unnecessary presence. Clinicians were allowed due independence and space to be able to perform their other duties.

*The supervision was OK because they were not stressing. You know we have our other duties; so if it is done frequently it would interrupt our duties but at least the time frame was OK…* (**C)**.

Moreover, the clinicians, patients and caretakers in the study had a relationship prior to the intervention. Patients in these groups were people the clinicians had been treating for a while. That existing relationship helped the organization and facilitation of the meetings since participants were not strangers to each other.

#### Perceived barriers for the meetings

Study participants observed a few challenges with regarding the location of the meetings, organization and facilitation of the meetings as well as participation. Location of the meetings at the regional hospital level was considered not conducive for attainment of the intended purpose of the FAPII. Health facilities in Uganda are graded beginning with the national referral hospitals at the top, followed in descending order by regional and district referral hospitals; health sub-district or health center four (HC IV), HC III, HC II, and HC I at the village level. Other than the government owned health facilities, the health care system also includes facilities owned by non-governmental as well as private agencies and individuals. Whereas the FAPII was conducted at a government owned regional referral hospital, the majority of the study participants suggested that decentralization of the intervention holds the key to its success. They advocated for devolution of the FAPII meetings to the lower level facilities like health centers IV, III and II to make the intervention more accessible.

*We should take it further down to our small or lower health centers like HC III because many people go there for medical services. …if these meetings get there, we shall capture many patients so that they get medication with the help of their family members* **(CT)**.

Some participants suggested that this intervention should be rolled out even in the non-government health facilities. Some even mooted the idea of moving the meetings away from health facilities to the communities and target other key stakeholders besides patients and caretakers, like the traditional healers and religious leaders. Drawing from their experiences in the FAPII meetings, they observed that the decentralization strategy would yield several benefits: (1) reducing patient numbers in the referral hospitals and also re-distributing clinical workload, which would likely improve the services provided since the clinicians would have fewer patients to attend to and therefore be able to provide more comprehensive mental health services including aspects like psycho-therapy; (2) increasing service uptake and reducing hospital visit fatigue by extending the continuum of mental health services from the health facilities to the community level targeting even the informal sector players; (3) enlisting greater involvement of patients’ caretakers in management of their mental illnesses by rendering it less costly in terms of time and money; and (4) improving awareness and knowledge of mental illness.

*Our meetings should not be used in hospitals (only) because by the time someone goes to the hospital he/she has known what he is suffering from. So they should be used in churches and places of traditional healers because people with mental health problems find it easier to go to them than health facilities especially during the early stages of the disease. In this way we shall (also) be able to show them that church alone is not enough without medication* **(CT).**

Notably, however, a few participants especially clinicians contended that the FAPII should be exclusively implemented in hospitals initially since the hospitals are more equipped with the necessary resources and personnel compared to the lower level facilities. They observed that once such services were well established at a referral hospital, sensitization would then be done at lower health facilities and the community to raise awareness about services available at the referral hospitals. They also suggested a blend of community support, and inpatient and outpatient services, noting however, that availability of resources for the strategy may be a challenge.

*The meetings should be in the big hospitals because that is where all the resources especially medicine are concentrated… ***(C)**.

The clinicians noted a language barrier; the main language of communication during the group meetings was Luganda, yet some group meetings included participants who were not natives of the area and were not proficient enough in speaking the language.

*The research was in the central but what I saw was if members of the group had a problem with the language, participation became difficult ***(C)**.

Some clinicians occasionally found it difficult to attend and facilitate meetings due to competing demands in terms of workload at the hospital and other social obligations. Clinicians’ workload issues were primarily attributed to understaffing in the health facility. In terms of social obligations, it so happened that some clinicians who initially committed to fully take part in the intervention got caught up in emergencies of life, rendering them unable to honor their commitment.

*Some people had other problems somewhere and they could not be able to make it as expected ***(C)**.

Furthermore, some clinicians considered the money budgeted to compensate them for their time to be insufficient; hence they could not fully participate in the intervention. *Maybe the clinical work load was part of it. But some (of us) were not happy because the money was not enough ***(C).** Relatedly, the project’s standard operating procedures (SOPs) included a provision to refund transport money to the participating patients and caretakers. Nonetheless, those participants had to first raise the money to transport themselves to the meetings and then get the refund. This was challenging for some because the majority of patients with SMI and their care takers were drawn from poor households and some therefore faced difficulty in raising money for their transport to the meetings.

*The first thing is transport; raising money for our transport is a challenge. For example if they stop giving us this facilitation for transport, we will end up not coming for the family meetings ***(CT)**.

### Perceived acceptability of the FAPII

Two broad sub-themes were evident in the data: (1) potential incentives for joining and remaining active in the intervention, which referred to the positive perceptions and experiences of the group meetings, and, (2) potential disincentives to joining and participating in the intervention, which referred to the negative perceptions and experiences of the intervention. The former were much preponderant.

#### Potential incentives

Most of the study participants (patients, caretakers and clinicians) perceived the scheduling and timing of meetings, which involved consensus building as conducive. Morning meetings left participants with ample time to continue with other matters later in the afternoon, including medicine refills at the clinic. The one-month interlude gave participants especially patients enough time to; rest, prepare for the next meeting, reflect on the topic and not forget what was discussed, and engage in livelihood activities – among others.

*The time and date were perfectly selected because we would begin with the meeting in the morning, and then in the evening, I conclude the day with other engagements. I prioritized morning meetings over other issues* **(CT).**

*The frequency enabled proper arrangement for the next meeting since it was once a month. We were also able to rest before the next meeting* **(P).**

Study participants generally liked the composition and process of meetings, especially the participatory approaches. The semi-circular sitting arrangement enabled inter-personal interaction within groups. Meetings were flexibly facilitated, allowing all members of the group the opportunity to contribute to the decision about topics and group management, including decisions on what to discuss and who to chair a session. If there were two opposing views on an issue, it was put to voting. Election of group chair from within the groups was considered important to participants as it empowered them by enabling them to learn from one another in groups and also built their self-esteem and respect.

*We would choose the point of discussion; we would call for suggestions; the things that are bothering you. You may find that what is bothering you is the same as what is disturbing us. Then we would go with what is voted by most people. For the chairperson, we would sit again and choose amongst ourselves at the end of every meeting ***(CT)**.

Most study participants considered the group size (10 to 17 people) as appropriate. It was small enough for all participants to know one another, maintain confidentiality of matters discussed in a group, and participate in the discussion. From a clinician’s point of view however, the group size could have been increased to allow more people to benefit from the intervention. The group size should in any case never exceed 25 people as larger numbers would render the groups difficult to manage.

*The number was good - small and manageable for a group discussion to give everyone the opportunity to share and contribute about the topic within the stipulated time… for this number everyone would come prepared knowing that each person would contribute and it helped them ***(CT)**.

*The number is OK; the group of seventeen is OK. If they are to add on it shouldn’t exceed a group of 25 because that would go out of hand and you would expect … problems ***(C)**.

The perception that the meeting offered a safe environment for sharing personal experiences and information encouraged participation. Participants were confident that the personal information they shared helped one another, yet could remain confidential. This was in contrast to their going to traditional and other alternative healers because often when a patient uses multiple healers, it becomes difficult to keep personal information private.

*It is really hard to find in the community what a patient shared here. Unlike before when I had just gotten ill, I moved from one traditional healer to another and the whole community got to know that I had run mad ***(P)**.

*At first, I was afraid but later I realized that … even if we shared, those people wouldn’t take it outside… ***(CT).**

The anticipation of knowledge and information from the intervention inspired many. Clinicians considered their selection to be part of the meetings as a great opportunity for learning. They had always wanted to learn more about their patients’ lives outside the health facility because they considered such information important in the management of the patients but time in the clinical setting was always not enough.

*I really wanted to know much of what was happening in these families because we rarely had much time to talk to them when they came for drugs; we would give drugs to them and then they go away but at least when you are there (at meetings) you can listen; a lot of things that you didn’t know ***(C).**

Caretakers embraced the meetings out of eagerness to know more about the mental illness in their families. They wanted to learn how other people were coping; so the opportunity to meet other people in similar situations was a motivation for them to join and continue with those meetings.

*When we heard about it… I was so excited about it and so was my son when I told him about it. We wanted to know so much about how other people manage when they have the illness. That is why we have been coming for the meeting consistently… ***(CT).**

Putting together clinicians, patients, and caretakers selected from the same clinical setting encouraged participation in the meetings since they already had established some kind of working relationship. The purposeful recruitment of patients and caretakers who already had established contacts with a mental health facility and also likely shared positive beliefs about mental illness contributed to the enthusiasm exhibited by participants in the meetings. Enlisting caretakers who were perceived by the patients to be the most involved in their care brought on board people with already tested commitment.

*The first is that, it was my mandate since he was my patient and my young brother; I was responsible for him whenever he got any problem; I had to support him because he could also support me* (**CT)**.

#### Potential disincentives

A perception of the long distance from participants’ homes to the meeting venue featured as a possible discouragement. The participants’ concepts of long distance and time were not clear in the group discourse. However, some respondents, especially caretakers, indicated that they travelled long distances to get to the meeting venue and spent a long time in the meetings although it did not necessarily jeopardize their morale.

*There was travelling for long distances yet we had other responsibilities; some other activities would be at a standstill but we still came with the patient to continue knowing more about this illness ***(CT)**.

As previously alluded, some respondents particularly clinicians were unhappy about what they perceived as the “limited reach of the intervention”. They felt that many more people should have been included and the information shared made more widely available in the intervention.

*These family groups; the thing I didn’t like was involving only a few people. They needed to give a chance to other people because the families benefitted from the program… ***(C).**

The heterogeneous constitution of some groups in terms of education and personal character sometimes hampered free group discussion. There were challenges in balancing the group discussions when the education level of participants was starkly varied because participants with limited education tended to have some difficulties following the discussion. Moreover, while some participants were too quiet in character, others were too talkative, which made it difficult for the chair to effectively steer the discussion, sometimes hampering the timely finishing of meetings.

*There are others who over-talk, the patients and even the family members; you would say it is time when they felt they still wanted to continue. So some felt like they needed more time while others wanted the meeting to come to an end. ***(C)**.

Whereas most participants reported that the selection of group leadership was participatory, a few reported that chairing of meetings in some groups was not strictly participatory and rotational. Some groups allowed the re-election of the same members to chair more than one session, much to the displeasure of some participants. Moreover, patients were not given the opportunity to chair sessions in some groups.

### Perceived impact of the FAPII

Three sub-themes emerged that suggest interrelated ways in which the intervention was perceived to have impacted patients, their families, and clinicians including: knowledge and information about mental illness; social networking and dealing with psychosocial issues of mental illness; and patients’ quality of life.

#### Knowledge and information about mental illness

Most of the caretakers and patients had limited knowledge of mental illness previously, which was largely changed by their participation in the intervention. The meetings culminated in new perspectives on mental illness, especially with regards to its cause and treatment. For instance, participants realized that mental illness is not caused by witchcraft and curses.

*I said in one of the meetings we decided to talk about the causes of mental illness in particular. So when we talked about the real causes of mental illness they realized that it (witchcraft/curse) has never been the cause* **(C).**

Participants understood that mental illness can be long-term and can be treated but through a gradual process; that through medication adherence, combined with counseling and social support, patients can get better and lead productive lives. Patients and care takers realized that mental illness was like any other illness, which emboldened their medical help seeking behavior. The patients realized significant improvement in mental health with medication unlike when they visited traditional healers where they stayed for extended periods and spent a lot of money but often registered little or no improvement.

*We first visited a number of traditional healers who took a lot of our money and I did not get any change at all. This drained my money and energy a lot. People could tell me to my face that I look like a mad person. But when I came to the hospital (and joined the group), I spent little yet I and the people I live with could notice a difference ***(P)**.

Patients and caretakers got more informed about their rights and responsibilities, which empowered patients to claim their rightful space in the public arena, for example by considering contesting for leadership positions and participating in other community activities. Patients’ quality of care improved; caretakers, for example, were enabled to pick up on early warning signs of mental illness onset/relapse and take appropriate and timely action.

*We were taught how to handle our mental state by adherence to medicine and (minding about) social inclusion… This is because we used to fear people and isolate ourselves and society could care less about us and in case of any social interactions they would exclude us ***(P)**.

*Through these meetings… I can now differentiate mental illness from other illnesses by looking at the symptoms and what is needed to treat the mental illness ***(CT)**.

The importance of a family in the provision of primary care and in the greater patient treatment schemes was highlighted. This contributed to improvement in the relationship between patients and their family caretakers. There was specific evidence of positive attitude change among individuals with mentally ill spouses. Family relationship was otherwise largely strained by the mental illness of a family member that is commonly known to stress the financial and emotional components of the affected families. The more harmonious family relationships enabled caretakers to care better for their patients since they could focus more on their patients and less on family relationship problems.

*As a caretaker, it has added a lot to me. Our relationship is now good, we can talk and I can advise him accordingly; I now know how to rebuke him when he makes a mistake… ***(CT).**

*My husband now can prepare a meal if he realizes that I am not well. Before, he didn’t understand and would force me to wake up and prepare food. When we started these gatherings, he understood the condition I was going through due to medication and he started to help me… Now he even allows me to pay someone to help with the laundry ***(P).**

Participants were further enlightened on the family’s important role in protecting the patient against stigmatization and discrimination. This impact even extended to the other family members as well as the members of the wider community who were not part of the intervention. This happened when participants in the meetings shared the knowledge thus acquired with other family and community members back home. Community members also changed after observing and appreciating the positive changes in patients’ lives.

*We normally share what we have learnt from these meetings and it is through this sharing that I noticed a behavioral change among my people at home; they are not hostile as they used to be. They also make it a point to include me in all the community activities like village meetings among others… The attitude of my family members and even the people in the whole village changed… ***(P).**

#### Networking and addressing psychosocial issues of mental illness

The meetings provided an appropriate platform/safe space to discuss and learn how to cope with the various non-medical challenges occasioned by SMI. The perception of a safe space enabled patients and caretakers to share feelings and unload emotions, which enhanced their connectedness. The meetings were a source of encouragement, psychological support, unity, and social network.

*This was a source of relief in my life since there are things that can’t be told in the community but when we come for these meetings … one has the freedom to air out what he or she is feeling. This is because you are narrating to people with whom you share similar challenges. This calms you down… These meetings made me understand that there are people with similar challenges like me…* **(P).**

Clinicians understood better the psychosocial problems faced by patients with SMI and caretakers in the environment where they live. They noted that some of their patients had been on pharmacotherapy for a long time without registering significant improvement, possibly due to the narrowness of the approach hitherto used to treat them. The meetings therefore alerted them of the need to learn and adopt new approaches to managing mental illness that combine pharmacotherapy with some psychosocial support in real time.

*The number one reason was, you realize that we have been given medication, western tablets and injections since Jesus was born and this has not reduced the number of patients with mental illness. I think we need to take another action… This intervention provides an opportunity to see what difference the use of psychosocial interventions would show ***(C).**

Clinicians noted that the caretakers rarely featured in their patients’ treatment and care plans previously and the intervention helped to bridge the gap. By sitting together in the meetings; clinicians, patients, and caretakers shared experiences and information, and were able to understand one another, which strengthened their relationship. Patients and caretakers were able to see the non-formal side of clinicians that they never witnessed in their regular visits to health facilities. Patients and caretakers felt freer to consult the clinicians because being part of group meetings together helped to break the barriers that previously existed in regular clinical encounters.

*Yes, I now seem to be closer to some of my patients and their relatives … At least now they come and consult me differently… I seem to have now changed from being just a health worker to (also) being a relative, family member, or family friend ***(C).**

*First of all the idea for the patients, caretakers and health workers to meet was very good … I started seeing them in a different way; that they are humane, and I really liked that ***(CT).**

#### Improvement of patients’ quality of life

The discussion about income generating activities during the meetings was insightful; patients realized that being mentally ill is not a total barrier to engaging in productive economic activities. It helped patients to initiate saving and income-generating activities. Indeed in the course of the intervention, more patients reported engaging in various economic activities to earn income, which helped to improve their living conditions.

*The issue of saving has helped me because since I got sick, they told us that when you start working like doing piggery, or having a grocery you have to save from your profits. It has also helped to know that mentally ill patients… should not be discriminated against* **(P).**

**…** *one of the topics was, ‘How do we improve the livelihood of families when there is a person with mental illness, including the livelihood of the person with the mental illness himself?’ … we talked about how to earn income from rearing chicken, keeping pigs, cattle, and (crop) farming. So some of them started to be more active …* **(C).**

By the end of the intervention, the majority of patients reported far less severe symptoms, were more involved in the activities of daily living, and were more socially competent overall. This positively affected the way they related with family and other community members. The understanding that mental illness challenges were not peculiar to them and the safe social space that prevailed in the meetings galvanized the self-confidence and self-esteem of patients and caretakers.

*Most of the abnormal things they used to report had reduced… the way they communicate, and the way they behave towards them, all had improved. The families were even involving them in some activities at home not like in the past… ***(C).**

*I believe in myself more. That is why I am even able to speak with you now… before this kind of treatment, I spent a year without talking. This was because I did not have what to say even if it was necessary. I now have confidence in what I do and even when I move around the community. More so I now engage in detailed conversations with the people I interact with… ***(P).**

## Discussion

We explored perceptions of mental health service providers and users regarding the feasibility, acceptability and impact of a family psychosocial intervention for persons with SMI in the Ugandan context. The intervention was depicted as feasible in light of current features such as: training of group facilitators, field support and supervision, prior relationship between participants, and convenient timing of meetings. Also, the perceived barriers were generally assailable. Acceptability of the FAPII was largely perceived as good in view factors including: anticipation of knowledge and information about mental illness, structuring of meetings, confidentiality of information, and appropriateness of choice of participants and venue. However, acceptability may be compromised by heterogeneity of groups and long distances to the meeting venue. Impact of FAPII was majorly felt in the following domains: provision of knowledge and information about mental illness, addressing psychosocial issues of mental illness, promoting social networking and bonding, and improvement of patients’ quality of life. Modification of the intervention by decentralizing it would greatly enhance all three dimensions – feasibility, acceptability, and impact.

### Perceived feasibility

The sub-theme of perceived enablers highlighted the major factors that were perceived to enable the intervention. The findings about the perceived contribution of training, field support, and supervision are harmonious with the report of a previous systematic review, which observed that to effectively implement family involvement in care, all members of a clinical team should be trained and continuously supervised. The report cited diverse studies that underscored the significance of continuing supervision and encouraging attendance in facilitating clinicians’ implementation of work with families while the lack of access to adequate supervision and training had the reverse effect [23].

Concerning the sub-theme of perceived barriers to the intervention, results highlighted several issues that, although largely surmountable, could jeopardize the feasibility of the intervention and ought to be addressed in future related interventions. The current model of delivery whereby the intervention is based at the regional referral hospital worked in the test phase but is fraught with challenges that may jeopardize its intended purpose beyond the current phase. Results, mainly reflecting perspectives of service users, instead highlighted a litany of advantages that would accrue from a decentralized model of delivery of the intervention and render it more feasible. Notably, among the proponents of a decentralized model of delivery of the intervention, a small section argued for a community-level model for delivery of the FAPII targeting the informal sector actors including traditional healers and religious leaders, which were reportedly popular ‘first ports of call’ when people are seeking care for SMI. Recourse to traditional (and faith) healers as the first line of treatment for mental illness has featured in multiple reports about diverse settings including Uganda [7, 24–28]. Up to 80% of patients found in Uganda’s mental hospitals will have been to a traditional healer previously [7]. Participants in the current study who advocated targeting the informal actors contended that the strategy would not only bring more people on board but also demystify those informal actors in the treatment of mental illness by enlightening the communities on their limits regarding mental illness. This is consistent with the results of a previous systematic review of studies in the Middle East where it was noted that a collaboration between traditional healers and mental health professionals may have a positive outcome since the affected families may feel more comfortable sharing their concerns and accepting the intervention involving those non-professionals [29]. A related recent study also conducted in central Uganda [25] noted the need for responsive interventions to balance the reality of the high use of traditional and faith healers, alongside evidence-based mental health care. The Uganda Ministry of Health has already attempted to formally work with traditional healers and according to their experience, many traditional healers are getting sensitive to the unique mental health needs of their patients and do refer such patients to health facilities for appropriate treatment as a result [30].

Heavy clinical workload apparently due to understaffing in the mental health clinic was a key impediment to clinicians’ participation in the intervention. The inadequacy of mental health specialists has been previously noted to seriously curtail access to effective mental health services in Uganda [7, 28]. Although staffing levels in the health facilities are outside the purview of the intervention, the possibility of recruiting more clinicians into such interventions should be explored with the view to re-distribute the workload occasioned by the intervention. All categories of participants reported some competing demands that tended to conflict with their attendance at meetings. The adverse impact of competing demands on family caretaker’s ability to provide quality care has been cited in other studies [27].

Financial compensation was identified as a potential barrier to the feasibility of the intervention. Notably, although financial compensation for clinicians and transport refunds for other participants were provided for under the research project, they may not be viable under routine health care services when the FAPII is eventually rolled out. These financial issues are likely to surface in all such interventions in low income settings and the matters should always be clearly interrogated and expectations managed appropriately. The finding that highlighted financial constraint as a potential barrier to participation in the FAPII is consistent with other studies in Uganda and other low resource settings [25, 27, 28] which noted that long distance to, and lack of transport to health facilities associated with poverty impeded accessing professional mental health care. This issue underscores the need to decentralize delivery of the intervention which would likely minimize the cost of attending the meetings. It should be noted however, that decentralization may not necessarily translate into increased access to services unless issues like the perceived need for treatment [3] are addressed.

### Perceived acceptability

Results depicted largely good acceptability of the intervention, driven by several incentives. Eagerness to learn more about mental illness was identified as a major incentive for patients, caretakers and clinicians to participate in the meetings. Similar incentives have been previously documented for the different types of participants in the meetings [18, 19]. The perception that the meetings and their choice of venue provided a neutral platform where participants could share experiences with colleagues featured among the factors that enhanced the acceptability of the intervention. This finding is consistent with the results of a study that explored the feasibility of psychosis seminars in the UK, which reported that the participation was encouraged by the perception that the seminars provided an inclusive and safe space where free speech was unencumbered [19]. It also somehow adds credence to an observation made in a past systematic review that clinicians should “uphold the patient–professional alliance by addressing privacy concerns and by being mindful that patients do not perceive a loss of power due to having family involvement in their care” [23].

The group size of 10 to 17 people was considered appropriate to the majority yet others advocated larger groups to benefit more people. Some meetings were also notably dominated by particular participants, suggesting that deliberate effort should be made during the recruitment of participants to harmonize their characteristics. These two potential barriers to the acceptability of the intervention have been featured in a similar study in the UK [19]. The UK study, however, recommended a larger number of participants per meeting (more than 25) compared to our study (not more than 25), implying a need for prior local consultations in determining the appropriate size of meetings. According to the FAPII manual, the selection of meeting chairpersons was supposed to be participatory and rotational. However, that requirement was not strictly adhered to in some groups, which is portrayed as a potential threat to the acceptability of the intervention that should be paid due attention in future such meetings. This issue featured in another study where participants proposed a remedy of outsourcing people to chair the sessions although the report is alive to the potential cost implication of the strategy [19].

### Perceived impact

Our results show that a major perceived positive impact of the intervention was in the broad domain of knowledge regarding mental illness and its management, which translated into an improvement in the quality of care for the patients. A previous systematic review of studies covering patients with schizophrenia in the Middle East cited studies that had noted the positive impact of family intervention on knowledge levels among service users and caretakers [29]. The positive impact of family members’ improved knowledge of mental illness on their ability to provide care especially through timely seeking of health care for the patient has also been previously documented in a study in Uganda [25]. The FAPII holds great potential as an avenue to improve the quality of care that can be accorded to persons with SMI through the enhancement of knowledge of mental illness among mental health service providers and users.

The current study results revealed a perception that the intervention improved patients’ adherence to medication, quality of life and social functioning. Patients’ improved social functioning reportedly positively influenced the attitude of family and community members, which in turn enhanced the quality of interaction between patients and the other groups. Improved compliance with medication and apparent general reduction in social impairment following family psychosocial intervention were previously reported in systematic reviews concerning the intervention with patients of schizophrenia and other schizophrenia-like illnesses [12, 13, 29]. The importance of good quality of relationship between patients and other people to the former’s overall quality of life has been previously noted whereby having good contact between family and friends was significantly associated with better quality of life for persons with SMI [31]. Furthermore, the current results indicate that the intervention provided an appropriate platform to discuss and address the various non-medical psycho-social challenges occasioned by SMI; patients and caretakers learned to cope with mental illness and its associated challenges; clinicians were enabled to explore the psychosocial problems faced by patients with SMI and their caretakers in a non-clinical setting, which altogether enabled them to learn and adopt new approaches to caring for people with mental illness. Moreover, the meetings strengthened the relationship between clinicians, patients. and their caretakers, which is consistent with previous meta-analysis of family intervention studies [12]. The intervention also provided a platform for patients and caretakers to share feelings and unload emotions, which in turn enhanced their connectedness. The FAPII in essence promotes a holistic understanding of, and approach to the management of SMI.

### Study strengths and limitations

To the best of our knowledge, this study is the first of its kind to explore the feasibility, acceptability, and impact of the FAPII for SMI in the Ugandan context. It provides important insights into the potential of this relatively low cost intervention to improve care for persons with SMI in Uganda and possibly other low resource settings. Implementation of the FAPII in Uganda adhered to adapted, variously cited trialogue guidelines [18, 19, 32] and can accordingly be replicated in other countries in the region. There were, however, some limitations to the study that should be noted. Patients who lacked close family caretakers to participate with them in the intervention were excluded. This eliminated many patients in a setting where mentally ill persons are highly stigmatized and discriminated against and calls for possible re-definition of family so that even such patients can be brought on board. Some participants fell sick along the way and could not attend all the sessions but there was a provision for their re-admission, so this could not have gravely affected their participation. Individuals were required that for a person to qualify to participate in the intervention they had to be proficient in speaking the Luganda language but it later turned out that a few non-native participants who were not fluent in the language were on board. Such people found it difficult to fully participate in the discussions. Clear measures should be put in place to eliminate such people during recruitment. The topics discussed in meetings and the group facilitators were not necessarily the same across groups and sessions, which may have contributed to variation in intervention outcomes between groups. This is an inherent limitation in the existing trialogue guidelines. There is need to ensure consistency of facilitators across groups.

## Conclusions

The study clearly shows that the family psychosocial involvement intervention promises to improve the quality of care for persons with SMI in Uganda and other similarly low resourced contexts especially if a decentralized model is adopted for the intervention to be delivered at the lower levels closest to the communities. Mental health services will become more accessible when they are made available through the non-clinical settings of meetings that are less costly in terms of time and money and are offered in a more neutral and friendlier environment as opposed to the usual clinical setup. Knowledge of mental illness and its management will be enhanced among patients, caretakers, and clinicians who participate in the meetings as well as the wider community members that those participants interact with. The important role of caretakers in the management of SMI will be more widely harnessed and recognized. A more holistic approach to the management of SMI will be adopted by the mental health care providers such that the non-medical psychosocial aspects of mental illness take center stage. Overall, the quality of life and social functioning of persons with SMI will improve.

## Data Availability

The datasets used and/or analyzed during the current study are available from the corresponding author upon reasonable request.

## Abbreviations

C: Clinician
CT: Caretaker
HC: Health Center
ICD-10: International Classification of Diseases and Related Health Problems – Version Ten
FAPII: Family Psychosocial Involvement Intervention
LMICs: Low-to middle-income countries
MNS: Metal, Neurological, and Substance use
P: Patient
QUIP: Qualitative Impact Assessment Protocol
SMI: Severe Mental Illness
SOPs: Standard Operating Procedures
UBACC: University of California, San Diego Brief Assessment of Capacity to Consent
UK: United Kingdom
WHO: World Health Organization
